# A Model of Memory Linking Time to Space

**DOI:** 10.3389/fncom.2020.00060

**Published:** 2020-07-08

**Authors:** Hubert Löffler, Daya Shankar Gupta

**Affiliations:** ^1^Independent Researcher, Bregenz, Austria; ^2^Biology Department, Camden County College, Blackwood, NJ, United States

**Keywords:** neural memory, temporally precise spike trains, subthreshold membrane potential oscillations, phase coding, gamma-theta code, working memory, spiking neural networks

## Abstract

The storage of temporally precise spike patterns can be realized by a single neuron. A spiking neural network (SNN) model is utilized to demonstrate the ability to precisely recall a spike pattern after presenting a single input. We show by using a simulation study that the temporal properties of input patterns can be transformed into spatial patterns of local dendritic spikes. The localization of time-points of spikes is facilitated by phase-shift of the subthreshold membrane potential oscillations (SMO) in the dendritic branches, which modifies their excitability. In reference to the points in time of the arriving input, the dendritic spikes are triggered in different branches. To store spatially distributed patterns, two unsupervised learning mechanisms are utilized. Either synaptic weights to the branches, spatial representation of the temporal input pattern, are enhanced by spike-timing-dependent plasticity (STDP) or the oscillation power of SMOs in spiking branches is increased by dendritic spikes. For retrieval, spike bursts activate stored spatiotemporal patterns in dendritic branches, which reactivate the original somatic spike patterns. The simulation of the prototypical model demonstrates the principle, how linking time to space enables the storage of temporal features of an input. Plausibility, advantages, and some variations of the proposed model are also discussed.

## Introduction

In daily life, we can distinguish between temporal and spatial properties of our world. In the brain, the temporal as well as spatial properties of the world is largely represented by spatiotemporal patterns of neural spikes. However, an important question currently facing scientists is: How the temporal dimension of the physical world is represented in the brain? The representation of time-dimension is required for a successful interaction of the brain with the four-dimensional physical world (Gupta and Merchant, 2017).

All hitherto presented ideas use spatial properties related to temporal ones even if their relationship is not directly addressed. Larson et al. (2010) explicitly spread time components into spatial components of an input as they investigated the question how sensory systems recognize time varying stimuli by spiking activity. Their model consisted of a succession of end-to-end excitatory neurons (neuronal chain) in combination with STDP to preserve the temporal features of spike patterns via their spatial distribution. In the neuronal chain model, the sensory input activates the first neuron in a chain of neurons, following which the neighboring neurons were activated sequentially with a delay of 2 ms.

Several past studies of spatiotemporal patterns are based on the processing the temporal features of an input. Many of these studies address the association between a precise input spike train and a desired output spike train in a temporally specific manner (Gütig and Sompolinsky, 2006; Ponulak and Kasinski, 2010; Florian, 2012; Mohemmed et al., 2012; Sporea and Grüning, 2013; Memmesheimer et al., 2014; Albers et al., 2015; Guo et al., 2015). These studies (known as ReSuMe**—**remote supervised method) assume that the temporal features of the input come from distinct neurons. The localization of the input time-points at different neurons is a precondition.

Data from several recent publications (Düzel et al., 2010; Lisman and Jensen, 2013; Bosman et al., 2014; Axmacher, 2016; Häusser et al., 2016) have provided some empirical evidence that memory for sequences of events is supported by the precise timing of item-related gamma activity with respect to underlying theta oscillations of membrane or field potentials. This neural interaction, referred as “cross-frequency coupling,” between lower (e.g., in the theta range) and higher (e.g., in the gamma range) frequency oscillations is also involved in sensory, motor and cognitive brain processes (e.g., Masquelier et al., 2008; Lisman and Jensen, 2013; Gupta and Chen, 2016; Maris and Fries, 2016). Furthermore, Remme et al. (2009) showed that dendritic potential oscillations enable dendritic inputs to be globally integrated on spatiotemporal scale, which can help to control somatic spike (action potential). Empirical data have also shown that oscillations and their combinations play an important role in neural memory of temporal events (Düzel et al., 2010; Headley and Paré, 2017). Thus, it is noteworthy that a dominant network pattern in the hippocampus is a slow oscillation in theta-alpha frequency band (Buzsáki, 2002).

There is deepening interest in the temporal processing of information in auditory system involving interaction between slow and fast oscillations. Different oscillation frequencies, corresponding to different pitches of auditory inputs, generate different spatial patterns in the auditory brain stem chopper neurons (Schreiner and Langer, 1988; Bahmer and Langner, 2006; Bahmer and Gupta, 2018). Modeling studies further suggest that chopper neurons are involved in the transformation of a temporal pitch code into a place code (Wiegrebe and Meddis, 2004). The topographic organization of temporal response characteristics in the auditory system suggests that the transformation of temporal properties, namely frequencies, into spatial patterns is beneficial in implementing neural code of pitch and harmony (Langer, 2015).

On the basis of the above research, the question arises how oscillations enable the transformation of temporal features into spatial ones.

Especially the interaction between two frequencies of oscillations enables a localization process, resulting in time-points according to phase-shifts. Phase coding refers to the process of encoding spike timing in relation to the oscillation phase of SMOs and has been empirically established (Nadasdy, 2009, 2010; Lundqvist et al., 2011; Hasselmo and Stern, 2014; Maris and Fries, 2016).

In fact, there is growing evidence for phase-shifted oscillations in neuronal units or ensembles. Sinha and Narayanan (2015) have shown that the differences in spike phase, due to modulation in either ionic channels or the synaptic conductance within the same neuron may be significant and vary by much as by 100°. In this study, the phases varied considerably as a function of radial distance from the soma, enabling spatial localization. In another study, Stiefel et al. (2010) reported that inhibitory postsynaptic potentials in cortical neurons can considerably shift the oscillatory phase. Cholinergic modulations change the power of oscillation as well as the magnitude of phase shifts. At least two distinct types of models of network activity have been proposed: intrinsic resonance property-based models and circuit-based models (Lee et al., 2018). The modulations can be caused by intrinsic SMOs or [e.g., by a rhythmic inhibition Fries, 2005, 2009, 2015]. Theoretical work, by simulating dendritic oscillations as weakly coupled oscillators with cable, shows that stable phase differences can be maintained between SMOs at different dendritic branches (Remme et al., 2009).

Based on phase differences between SMOs in different dendritic branches, in the following section a model is described linking time to space by encoding the temporal pattern of a spike train from an input neuron into a spatial pattern of a memory neuron, where it is stored by STDP, thus allowing recall and retransformation from spatial into temporal pattern, everything happening in a single neuron following a single input train.

In the next chapter the structure of the model is described, illustrating the mechanisms of encoding, learning and recall, also showing the main equations, which underlie the simulations. The Results section presents detailed simulation data with particular focus on the alterations of dendritic and somatic processes, again during encoding, learning, and recall. The biological plausibility of the model and diverse specific points will be discussed, followed by a conclusion.

## Materials and Methods

**Figure 1:**
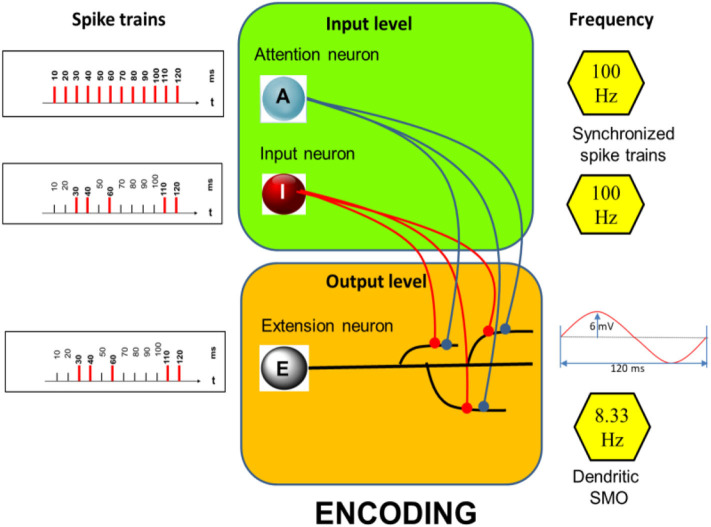
Encoding: Structure of the model with examples of spike trains **(Left)** aligned to a basic rhythm of 100 Hz (indicated on the right) including an example of a dendritic SMO of 8.33 Hz **(Right, Bottom)**. Here only three dendritic branches are shown while the simulations are run with 12 branches, each exhibiting subthreshold oscillations of the same frequency but different phases (which are equally distributed across the oscillation period). Neuron A and I are synaptically connected (filled circles) to all 12 dendrites.

The prototypical model**—**as outlined below**—**consists of an input and an output-level (Figure 1, inserts**—**green and orange). The input level comprises two neurons, the input neuron I and the attention neuron A. The activity of neuron A is considered a correlate of “attention” serving storage and recall. The output level consists of a single neuron, the extension neuron which comprises several dendritic branches with the particular feature that they all exhibit subthreshold membrane potential oscillations of the same oscillation period but of different phases.

Spike activities of I and A (Figure 1, left) are assumed to be synchronized by LFPs in the gamma band range (Rodriguez et al., 1999). Neuron I fires randomly but temporally precisely aligned to the spikes of the regular firing neuron A. Here their common basic rhythm is 100 Hz. The dendritic SMOs are in the theta band range (here 8.33 Hz, see inset of Figure 1, right, bottom). These values are in a biologically plausible range. Gamma frequency in our model can vary between 100 and 30 Hz and theta frequency can vary between 10 and 5 Hz without affecting the principle simulation outcomes. Only the number of storable time-points (12 in the example of Figure 1 simulations) depends on the relationship of theta to gamma frequencies. Low theta oscillations combined with high gamma oscillations allow most spikes to be stored.

### Encoding

**Figure 2:**
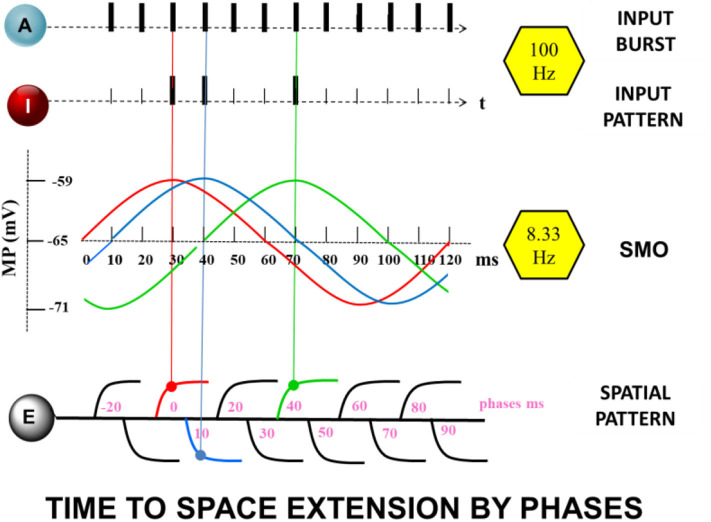
The “Time to Space Extension Process” A continuous spike train of the attention neuron A together with randomly appearing, although precisely aligned spikes of the input neuron I, coincide with maximum values of subthreshold dendritic oscillations of the extension neuron E at different dendrites (same color as the oscillations) leading to enhanced oscillations or increased synaptic efficacy (STDP).

The core of the presented memory model is the time to space extension process during encoding (Figure 2). It takes place in neuron E. Dendritic branches of E represent the spatial dimension. The transformation of input times into a dendritic location depends on the coupling of the gamma input-frequency with the theta SMO-frequency in the branches of neuron E. The dendritic branches serve as the coincidence detectors. Axons of neuron I and A connect to all branches of E and propagate spikes to them. Only a specific combination of synchronized inputs from I and A generates dendritic spikes in E. Additionally, a third component is necessary for a dendritic spike generation. The intrinsic sinusoidal SMO of a branch must be near the peak so that it is sufficiently excitable. Only the coincident inputs from A and I at the peak of dendritic membrane excitability at a branch lead to a dendritic spike.

Finally, the soma of neuron E accumulates the scaled potentials of dendritic spikes with scaled potentials of arriving inputs from I and A. All three membrane potential changes sum together to produce a somatic spike in neuron E. It is believed that the impact of dendritic oscillations on the soma is balanced out because of the distribution of phases between branches. Therefore, the effect of dendritic oscillations on somatic membrane potential are excluded.

### Learning

For the storage of inputs, a learning mechanism is required. In the proposed model, we consider two “unsupervised” mechanisms for learning, occurring whenever a dendritic spike in E is generated due to the coincidence of spikes from neurons I and A with a depolarization maximum of SMOs in the dendrites of extension neuron E. Gamma-range coincidence detection corresponds to the findings of Das et al. (2017), Das and Narayanan (2017) in hippocampal CA1 neurons, enabling to decode synchronous gamma-frequency inputs. Storage and recall can be achieved either by STDP and also by activity dependent alterations of the amplitude of the SMOs. For simplicity, the different dendrites of neuron E, are considered as functionally independent units not interfering with each other and not being affected by somatic spikes although each spike in any one of the dendrites generates a spike in the neuron's soma.

#### Learning by Spike Timing Dependent Plasticity (STDP)

The first strategy to store the spike pattern from input neuron I is based on the enhancement of synaptic weights. The choice of the initial synaptic weight is arbitrary, but it is interrelated to other neuronal parameters.

The synaptic weights between input neuron I and the dendrites of neuron E, set to W(IE) = 0.15, are kept constant, unaffected by the learning process. The relevant changes have to take place at the synapses from neuron A to neuron E because recall will be initiated via this pathway when the input neuron will be silent. Hence, the EPSP amplitudes from neuron A have to be sufficiently increased to compensate for the lack of the EPSPs from input neuron I.

The synaptic weights from neuron A to all branches of neuron E initially amount to W(AE)_ini_ = 0.40. In this model a single spike increases the synaptic weight to W(AE)_max_ = 0.55, (i.e., by about 37% of its initial value). This will increase further EPSP amplitudes which shall be sufficient to store the information of the timing of a spike input from neuron I in the corresponding dendrite of neuron E for later recall by neuron A. Such a potentiation seems biologically plausible. Remy and Spruston (2007) used a single burst of only five spikes at 100 Hz as stimulation and showed that hippocampal synapses potentiated robustly under this condition. LTP was triggered whenever there were dendritic spikes. Amplitudes of EPSPs could be robustly potentiated by 66% (from ~7 to ~12 mV).

Single somatic spikes do not significantly change the synaptic weights at dendrites. Since the proposed model is a single trial, involving a single spike burst, the propagation of somatic action potential will have a negligible effect on LTP.

#### Learning by SMO Amplification

In an alternative learning mechanism, the encoding occurs due to the enhancement of the amplitude of the SMO after dendritic spikes. This is consistent with the enhancement of amplitude of theta oscillations from cortical EEG recording in a working memory task in humans (Raghavachari et al., 2001) as well as with the findings that neuronal activities in the hippocampus change with individual theta phase in monkeys (Skaggs et al., 1996). Moreover, significant enhancement of oscillatory power observed during encoding has predicted subsequent recall. This effect has been found predominantly in the 4–8 Hz (theta) and 28–64 Hz (gamma) frequency bands (Sederberg et al., 2003). Ness et al. (2016) showed that local field potentials could indeed be utilized to characterize the properties and cellular distributions of active conductance.

In our model the SMO amplitudes are initially set to 6.0 mV. In those branches of neuron E in which a dendritic spike is generated and these values are enhanced by 50% to 9.0 mV which, again, is sufficient to store the information of spike times arriving from neuron I in spatially distributed branches of neuron E for later recall by neuron A.

### Recall

Both learning mechanisms allow the encoding and recall of the input spike train. Two out of three neurons, A and E, play a role in the recall process. Recall is triggered by a continuous spike train from A to E, synchronized at the same gamma frequency (100 Hz) as used during the encoding. During this recall, the input neuron is silent. The impact of the input neuron during encoding is substituted by enhanced synaptic weights from neuron A. Furthermore, during recall, the dendritic branches fire at time-points corresponding to the input spike train that was initially encoded. Persistence of phase-shifted SMOs in the dendritic branches together with enhanced synaptic weights would then result in the recall spikes at dendritic branches. Dendritic spikes generate somatic spikes in E matching the output during the encoding process. It should be noted that the recalled spike train evokes the somatic spike train, generated by the input spike train, not the input spike train itself. The input spike train is recalled only, if it reproduces an equivalent somatic spike train during encoding.

### Simulation

A major goal of this study is to show that a single neuron would be able to store temporal properties of an input by the spatial pattern of dendritic branch activation. The current simulation study uses simple mechanisms, such as theta-gamma-oscillations combined with LTP, which is implemented in a small SNN.

Gamma oscillations are represented in the rhythm of spike inputs. Theta oscillations are explicitly modeled by phase-shifted sine functions f(OP):

(1) $$\begin{array}{l} f^{\prime }\left(OP\right)=h*sin\;\left(0.002*\pi *fq*\left(t-j*ph\right)\right) \end{array}$$

where *h* = 6.0 mV is the oscillation amplitude, fq = 8.33 is the oscillation frequency, *t* is the time in ms and ph = 10 ms is the steps size of phases, multiplied by *j* = −2 to 10.

Referring to the model of Legenstein and Maas (2011), each spike input to the dendrites of the extension neurons causes an EPSP modeled in the form of an alpha function:

(2) $$\begin{array}{l} \text{f}\left(\text{EPSP}\right)=k*W*\delta t*g^{-\frac{\delta t}{\tau }} \end{array}$$

where *k* = 39, *g* = 2.0, and τ = 1 are constants and *W* is the synaptic weight (either from I to E or from A to E: the initial W(IE)_ini_ = 0.15 and initial W(AE)_ini_ = 0.40. δt is the time difference to the EPSP onset.

SMOs and EPSPs sum up, which generates a dendritic spike whenever a threshold φ^dend^ = −48.7 mV is reached. The branch spike potential (BsP) is again modeled in form of an alpha function:

(3) $$\begin{array}{l} \text{f}\left(\text{BsP}\right)\;=k*\delta t*g^{-\frac{\delta t}{\tau }} \end{array}$$

where *k* = 40, *g* = 2.0, and τ = 1 are constants with δt as the time difference to the dendritic spike onset.

The total branch potentials are summed up by the SMO potentials, the local EPSPs and spike potentials.

EPSPs and spikes propagate to the soma, scaled by different weighting factors, assuming active conduction of dendritic spikes without attenuation (u_B_ = 1.0) and passive, electrotonic propagation of EPSPs with decay to u_pass_ = 0.08.

Alterations of somatic membrane potential MP introduced by an individual dendritic branch are calculated by:

(4) $$\begin{array}{l} \text{MP}=\text{EPSP}*u_{pass}+\text{BsP}*u_{B} \end{array}$$

Time delays and different distances of the different branches to the soma are not considered. Dendritic SMOs potentials are without any effects on the soma.

**Figure 3:**
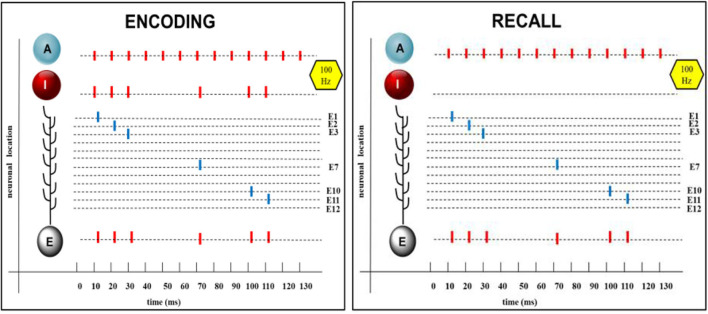
Encoding and recall of a randomized spike pattern. From **Top** to **Bottom**: A: Continuous spike sequences of the attention neuron, which are the same during encoding and recall. I: randomized spike pattern of the input neuron, shown here with six spikes aligned to the spikes of the attention neuron. Single dendritic spikes at different synaptic branches (E1 to E12), appearing during encoding and also on recall, in both cases, summing up to produce a spike train in the soma of the extension neuron E, which is the same as in the input neuron. Short time delays (2 ms) are introduced between onset of the input spike and of the action potential in E.

Somatic spikes emerge if the somatic potential exceeds the somatic spike threshold S and are represented as abrupt increase to +30 mV for 1 ms followed by a transient hyperpolarization with exponential reduction. Input spikes, dendritic and somatic spikes are represented by bars as shown in the summarizing Figure 3.

The simulations are calculated with time resolution of 1 ms.

The simulation program was written by the author (HL) in Python 3.7 (Supplementary Materials - Datasheet 1 and Table S2).

Parameters for somatic, dendritic, synaptic and oscillation properties are shown in Table S1.

## Results

The model has been tested with input trains of random numbers of input spikes at randomized times, however, adjusted to the 100 Hz gamma rhythm of the spiking of the attention neuron. Test runs have been made with spike inputs and recall during a full cycle period of the dendritic SMOs (120 ms). In this period the attention neuron fires a continuous sequence of 12 spikes.

For encoding and learning, additionally a randomly chosen number of up to 11 spikes from the input neuron arrive at the dendrites of the extension neuron. These input spikes are synchronized with the spike times of the attention neuron but appear at random positions. In any case, due to the above described mechanisms, (i.e., superposition of EPSPs from the input neuron and the attention neuron in correct phase with the SMOs at the dendrites), (i.e., in their maximum, the input patterns also appears at the extension neuron).

Recall is initialized by the application of a spike burst from the attention neuron A while the input neuron I is silent. Due to the learning mechanism, (i.e., increased EPSPs by STDP or enhanced SMO amplitudes, the same pattern as during the input phase reappears at the extension neuron).

This was confirmed by numerous (100) trials with randomized input patterns each showing an exact reproduction of the initial input pattern in the extension neuron after learning and recall. An example is shown in Figure 3.

Details of functionally relevant alterations of dendritic and somatic mechanisms during encoding and learning and how these are enabling correct recall are shown in the following sections.

### Encoding

**Figure 4:**
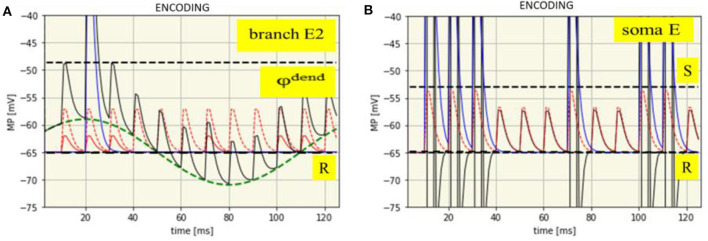
Time course of membrane potentials during encoding of the spike pattern IP1: dendritic in branch E2 **(A)** and somatic **(B)**. R: Resting membrane potential (-65 mV, black dashed line), φ^dend^: Threshold for dendritic spikes (-48,7 mV, black dashed line). Green dashed line: SMO (phase: -10 ms). Red dashed line: EPSPs from neuron A. Red solid line: EPSPs from neuron I. Blue solid line: Dendritic spike potential appearing once at t = 21 ms. Thin black line: Course of the net branch membrane potential.

Dendritic processes: The net membrane potential of the branch E2 is the sum of the intrinsic potentials of SMO and EPSPs from local synaptic activation by neuron I and neuron A, eventually superimposed by a dendritic spike. The time course of all these potentials is shown in Figure 4A with an example from the synaptic branch E2 in which a dendritic spike is generated. This happens only in the maximum of the SMO where the sum of EPSP is strong enough to reach the threshold φ^dend^ = −48.7 mV for spike generation.

In other branches, dendritic spikes will not be generated at this time, even when the EPSPs are the same. The reason is that potential of the phase shifted oscillations is too low. Spikes in other branches will be generated when their oscillations are in their maxima, provided that there is again spike input from both neurons at the input level, (i.e., when in addition to the attention neuron the input neuron is also firing). In this example, already shown in Figure 3, this happens six times, always in the maximum of the oscillations. In different braches spikes are generated at different time-points due to their phase shifted oscillations.

Somatic processes: The dendritic spikes propagate to the soma together with EPSPs from all branches. However, as it needs the combination of both to exceed the threshold of spike generation the same spike pattern will appear at the extension neuron as received from the input neuron (Figure 4B).

### Learning

**Figure 5:**
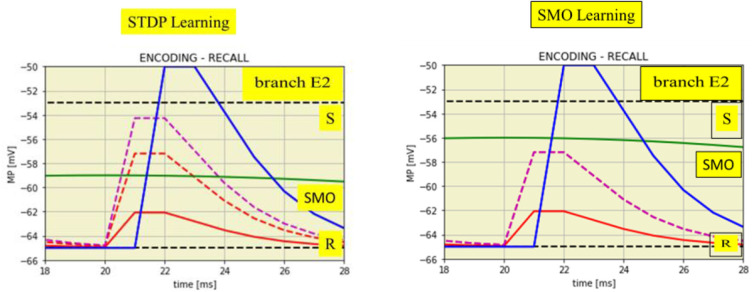
Time expanded cutouts of Figure 4A (from *t* = 18 to *t* = 28 ms) also illustrating two different learning mechanisms. **Left**: STDP by enhanced EPSPs on spike input from the attention neuron. EPSPs from the input neuron (two red lines) change to the enhanced EPSP from attention neuron (magenta dashed line). **Right**: increased SMO amplitude, from 6.0 to 9.0 mV, shown by the green line in comparison to the green line in the left figure.

Learning is implemented at the extension neuron either by enhanced EPSPs from the attention neuron or by increased oscillation amplitudes of the dendritic branches (Figure 5). These alterations, however, only appear at those dendrites in which a dendritic spike has been generated during encoding by synchronized input from A and I at the maxima of the dendritic SMO. In this way, the information about the temporal pattern of the previous spike input is stored, spatially distributed, in dendritic branches with accordingly altered properties to allow recall.

### Recall

The previous spike pattern from the input neuron can be recalled by a burst of spikes from the attention neuron while the input neuron is silent. The attention neuron thereby has to fire at the same gamma rhythm as during encoding and the spike burst should have in minimum a duration of the length of the theta cycle over which the maxima of the SMOs are distributed. This guarantees that each dendrite will receive a spike input from the attention neuron when its SMO is close its maximum. In this case, a single spike from the attention neuron can elicit again a dendritic spike, however, only in those branches in which the learning mechanism, as described before, have either increased the oscillation amplitude or enhanced the synaptic efficacy due to enhanced EPSPs. The one like the other is sufficient to compensate for the lack of spikes from the input neuron. Dendritic branches without these learning effects will remain subthreshold even when a spike from the attention neuron input hits the oscillation maxima.

**Figure 6:**
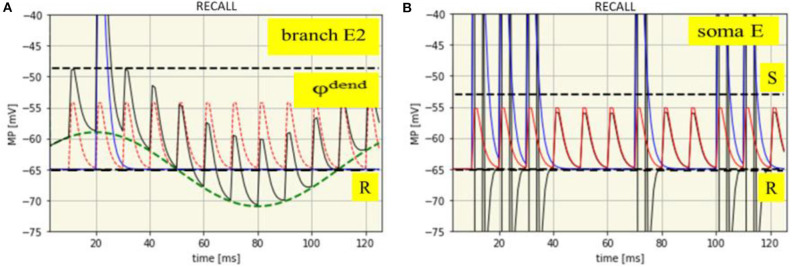
Time course of membrane potentials during recall of the spike pattern IP1: dendritic in branch E2 **(A)** and somatic **(B)**. R: Resting membrane potential (-65 mV, black dashed line), φ^dend^: Threshold for dendritic spikes (-48,7 mV, black dashed line). Green dashed line: SMO (phase: -10 ms). Red dashed line: EPSPs from neuron A. Blue solid line: Dendritic spike potential appearing once at t = 21 ms. Thin black line: Course of the net branch membrane potential.

Figure 6A shows how a dendritic spike is generated only by an EPSP from the attention neuron, in this example due to enhanced synaptic efficacy. This example is again drawn for branch E2 in which learning mechanisms have been introduced as illustrated in Figure 5. The same happens at all other branches with enhanced EPSPs or increased oscillation amplitudes but not in those branches without learning effects. The spikes will be generated in the same sequence as the learning mechanisms have been introduced in the different braches by spikes from the input neuron.

Nothing has changed with spike generation at the soma due to the propagation of dendritic EPSPs and spikes. Propagation of dendritic EPSPs alone cannot generate somatic spikes. This needs the contribution of dendritic spikes. Hence, dendritic spikes, together with dendritic EPSP will propagate to the neuron's soma, there generating a sequence of spikes which exactly reflects the previous input pattern (Figure 6B), just recalled by a gamma spike burst from the attention neuron.

## Discussion

The simulation of the current model demonstrates how temporally precise spike trains can be stored by the spatial pattern of dendritic branches even in a single neuron. This mechanism can precisely store temporal information about onetime presented single spikes via the interaction of neural oscillations. The spike trains can be just recalled by a continuous spike burst. The key feature of this model is the spatial localization of spike-timing, which is established by phase-shifted theta oscillations of excitability in dendritic branches in combination with gamma-aligned input patterns. This mechanism links the temporal order of spikes of the input to different dendritic branches, allowing the transformation of temporal properties into the spatial pattern of dendritic activation. The spatial localization of the timing information enables the storage of temporal properties by learning mechanisms.

### Other Approaches to Realize Linking Time to Space

Our prototypical model illustrates within a single neuron the general mechanism of transformation of time into spatial dimensions using cross frequency coupling of neuronal oscillations. The same approach can be adapted to ensembles of neurons. Analogous to the dendrites of a single neuron, an ensemble of neurons can work together as canonical microcircuits based on same principles. However, instead of the SMOs of dendrites, the somatic SMOs of ensembles vary in phases. The somatic spikes of single neurons correspond to the dendritic spikes in our prototypical model. The somatic outputs of the ensemble neurons have to be propagated to an additional output neuron, corresponding to the somatic output of the single neuron. A simulation trial of this alternative model-version has produced identical results. A bursting input by neuron A to the ensemble neurons reproduced an equal temporal pattern in the output neuron as was produced during the encoding process. Future simulation studies could reveal the effect of randomness in the spike trains or various oscillation parameters in neuronal ensembles on the spatiotemporal patterns.

Some further assumptions of our model can be changed without destroying its functionality. For example, the regular distribution of phases of SMO within spatial units can be replaced by random phases, if the number of dendritic branches was enhanced from 13 to 48. Preliminary simulations led to 96% right recalled spikes and 85% completely right recalled spike trains, using 1,000 random input patterns.

Moreover, oscillation-based conversion from temporal to spatiotemporal neural patterns can be also useful in the reverse direction. Since a temporally precise spike train can be represented by a more complex spatiotemporal neural pattern, a spatiotemporal pattern can be propagated to neuronal areas far apart from the origin by the corresponding simpler temporal code. The spatial part of the information can be rebuilt by synchronized oscillations between the area of origin and a target area. In summary, via oscillations spatial information can be propagated by temporal sequences, and these temporal sequences can be efficiently stored by transformation into neural spatial patterns.

### Dendrites in Information Processing in Perception

The assumption of dendritic branches acting as independent subunits to process the memory of spike trains appears to be feasible (Golding et al., 2002; Behabady and Mel, 2013; Bono and Clopath, 2017). The independence of dendritic processing allows dendritic spikes to play an important part in information processing since they significantly increase the probability of somatic spikes (Oesch et al., 2005; Polsky et al., 2009). According to the current prototypical model, a coincidence detector, comprising of A neuron along with I neuron (providing inputs from hierarchical sensory areas) and the phase of the SMO when it peaks, is responsible for dendritic spike, which then leads to the learning by STDP. This learning mechanism is responsible for encoding. Furthermore, coincidence detection will occur only at those dendritic branches, where the magnitude of phase shift with respect to the first or a reference dendritic branch is quantitatively equal to an integer multiple of periodicity of continuous input from neuron A. This integer multiple of periodicity of continuous input, called the integration period (Bahmer and Gupta, 2018), encodes the information about the individual spikes in the spike train during the learning stage. STDP, which increases synaptic weights on spatially distributed synapses is responsible for the recall. Continuous spike train, synchronized by the same frequency, which was used during encoding, is responsible for the recall of the initial input from neuron I in this model. Moreover, the learning during the encoding stage will increase the certainty in neural circuits given the knowledge about the source of input that is neuron I.

As discussed previously by Gupta and Bahmer (2019), perception is contributed by both increase in surprisal as well as increase in certainty given the knowledge about sensory object. In the present prototypical model, surprisal would be due to the presence of inhibitory synaptic input at individual dendritic branches. The inhibitory inputs would suppress some of the dendritic spikes, preventing a complete recall. Thus, some of the spikes during the encoding process could be subjected to suppression by inhibitory synapses via a random process given the certainty about sensory object. Since sensory inputs may control the activity of inhibitory neurons in the cortex, the addition of inhibitory synapses could endow the prototypical model the ability to process sensory information for more complex, cognitive functions. Moreover, it is therefore noteworthy that inhibitory synapses are shown to play key roles in cortical information processing.

**Figure 7:**
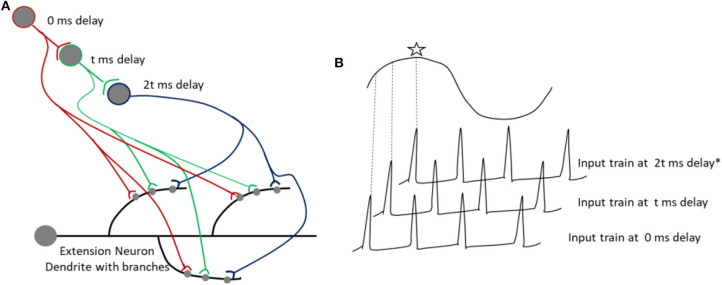
**(A)** Shows the arrival inputs at dendritic branches, after being processed in parallel circuits with varying number of synapses. The parallel processing allows a single spike to arrive after various delays at the dendritic branches. A synaptic delay (*t*) can vary from 0.5 to few ms. **(B)** Shows a spike coinciding with the peak of a SMO, indicated with an asterix.

During information processing in perception, spike trains can be produced by a single spike processed in parallel, which would arrive at multiple synapses between neurons I and E after various delays. Thus, the results of parallel processing can be encoded and recalled as a pattern of activation of dendritic branches. One such plausible mechanism is illustrated in Figure 7, wherein a single spike, processed in parallel, arrives at multiple synapses after various delays, which coincides with the peak of SMOs in a specific set of branches of dendrites, resulting in a spatial pattern. Processing of a single input in parallel circuits, as shown in Figure 7B, will result in multiple outputs, which will arrive at different dendritic branches after various delays, caused by synaptic transmission delay. Also note that a synaptic delay can vary between 0.5 and 5 ms. A spike is encoded as a spatial pattern, constructed by STDP induced at different synapses in a particular set of dendritic branches. During a recall, the same specific input as a result of parallel processing, coinciding with the SMO of dendritic branches, would be responsible for reproducing specific dendritic spikes. Thus, this proposed mechanism within the framework of current prototypical model can explain how a complex information about a simple stimulus (a single spike) can be temporarily stored in specific areas of the brain, where a specific parallel circuit configuration may be available.

### Duration of Storable Spike Trains

Short spike trains lasting up to 120 ms as used by our simulation may include the reaction time and reaction intensity of a new input without an adaption process. A memory of this initial part of information is relevant for the further information processing. However, the length of spike trains that can be stored by the proposed model is not limited on the length of a single theta oscillation cycle. This restriction pertains only if one uses a single neuron as bursting input during encoding (in our model by neuron A). Nevertheless, the additional part of a longer lasting input spike train can be stored by a second bursting neuron via its connections to the same (or a new) extension neuron. A temporal driven sequence of bursting neurons [e.g., by time cells (Eichenbaum, 2014) can encode and recall any long input spike train]. Moreover, using lower SMO- frequencies (e.g., delta) the duration of storable spike trains increases. If, for instance, the theta SMOs of our model are replaced by low delta (1 Hz) SMOs and the gamma aligned spike trains are replaced by low beta aligned (12 Hz) trains of about 10 spikes within one second can be exactly stored. Such delta-beta frequency coupling underlying sleep spindles is often associated with memory consolidation.

### Working Memory

The mechanisms of the model enable a single trial encoding of temporally precise spike trains by single neurons and their fast and simple retrieval via a non-specific input of spike bursts. These are requirements holding for a working memory, too. However, the working memory has been believed to be established by sustained neuronal spiking, triggered by external events. Yet, there is no necessity for sustained spiking activity of an input, if the contents of working memory can be reactivated immediately, which is possible with the proposed prototypical model. Indeed Stokes (2015) suggests, that a delay in mnemonic activity in the prefrontal cortex is not always critical for maintaining the continuity of working memory. It can be re-established when attention is directed to the task-relevant content. Furthermore, Lundqvist et al. (2016) and Fiebig and Lansner (2017) point out that without sustained spiking, energy would be conserved during inactive states and information is not lost when activity is disrupted, and attractors can hold multiple items in working memory. They extended an attractor network model for memory encoding and recall by oscillations. Experimentally, the authors observed gamma bursts for activation and reactivation of inputs. Similarly, our model proposes gamma bursts of the attention neuron accompanying the input for encoding and gamma bursts again during the recall. Gamma bursting is a general form of the activation patterns of neurons in the central nervous system (Cooper, 2002).

Because in our model the recall is generated by a bursting input from the same neuron (A), which was active throughout the encoding process, a further interesting option presents itself: The continuity of bursting yields original output of the input spike train, even if the input train itself is finished. Thus, the output of the terminated input is replicated as long as the bursting lasts. Even a complex input pattern within a group of neurons can repeat oneself as long as the burst continues. In addition, using SMO-learning instead of LTP, a properly tuned bursting input from any neuronal source can recall the stored input patterns and realize the working memory.

## Conclusion

Authors suggest that the realization of neuronal memory for temporal events is restricted to distributing temporal properties of events over spatially different units. The ubiquitous brain oscillations combined with the synchronization of neural activities can store temporal information as spatial patterns. Frequency, phase and amplitude**—**the three main characteristics of oscillations**—**can work together for this purpose. Significantly, the simulations presented in this study show that neural oscillations can allow time-dimension to be linked with the spatial dimensions in the brain circuits, which is important for the cognitive functions in interacting with the four-dimensional physical world. Moreover, simple spike bursts frequency aligned to the input events can serve as the trigger for retrieval as well as correlate for attention. The utility of the proposed memory mechanism for various brain functions, such as working memory is also evident. Preliminarily, our model is for now a theoretical hypothesis supported by computational simulations using physiologically plausible range of parameters. As a prototype model, it will be further enhanced by additional biological findings.

## Data Availability Statement

The datasets generated for this study are available on request to the corresponding author.

## Author Contributions

HL and DG contributed conception and design of the study. HL simulated the model and wrote the first draft of the manuscript. DG wrote sections of the manuscript. Both authors contributed to manuscript revision, read and approved the submitted version.

## Conflict of Interest

The authors declare that the research was conducted in the absence of any commercial or financial relationships that could be construed as a potential conflict of interest.
