# Supplementary material for: A Model of Memory Linking Time to Space
Source: Front Comput Neurosci. 2020 Jul 8;14:60. doi: 10.3389/fncom.2020.00060 (PMC7360808; doi:10.3389/fncom.2020.00060)
Supplement: Supplementary file 1 [file Data_Sheet_1.PDF]

```
# A Model of Memory Linking Time to Space

# Hubert Loeffler, Daya Shankar Gupta


import pdb#; pdb.set_trace()

import sys # to exit of the programm

import  random # random

import inspect # line number

import math

import numpy as np

import matplotlib.pyplot as plt # plot graphics


# =====================================================================================

def interrupt():

    programPause = input("Press the <ENTER> key to continue...")


#"""""""""""""""""""""""""""""""""""""""""""""""""""""""""""""""""""""""""""""""""""""""""""""""""""""""""

def lineno(): #line number

    return inspect.currentframe().f_back.f_lineno


#"""""""""""""""""""""""""""""""""""""""""""""""""""""""""""""""""""""""""""""""""""""""""""""""""""""""""

# dLTP

def dLTPsub (n,b,W,Wm):

    W[n][b][1] = Wm[n][b][1]

    return(n,b,W,Wm)


#"""""""""""""""""""""""""""""""""""""""""""""""""""""""""""""""""""""""""""""""""""""""""""""""""""""""""

# Oscillation amplification

def dOAsub (n,b,TOH):

    TOH[n][b]= TOHmax   #INPUT '# amplified oscillations at branches of neuron E after a dendrritic spike reached by encoding)

    return(n,b,TOH)
```

```
# ==================================================================================
# Simulation
def simulation (dLTP,dOA):
    for T in range(3,nT):
        n=nI+1
        for n in range (nI+1,nN+1):
            b=0
            while b < nB0:
                if C[n][b] == 0:
                    b = b+1
                O[n][b][T] = TOH[n][b]*math.sin(0.002*ThetaFq*(T-TOPh[n][b])*math.pi)
                Oabs[n][b][T] = TOH[n][b]*math.sin(0.002*ThetaFq*(T-TOPh[n][b])*math.pi)+gR
                for s in range(0,len(C[n][b])):
                    x = C[n][b][s]                          # position in C
                    if ST[x][0] is not 0:                   # exit, if branch without synaptic inputs (=0)
                        for z in range(0,len(ST[x][0])):        # e.g. [10,30]
                            if ST[x][0][z] is not 0 and T > min(ST[x][0]):
                                y = T-ST[x][0][z]


                                if y > 0:
                                    Es[n][b][s][T] = Kabs_d * W[n][b][s] * y * G1 ** (-y)
                                    Esabs[n][b][s][T] = Es[n][b][s][T] + gR
                                    E[n][b][T] = E[n][b][T] + Es[n][b][s][T]


                Eabs[n][b][T] = E[n][b][T] + gR


                # dendritic spike generation
                if (E[n][b][T] + O[n][b][T]) > (S[n][b][T]-R[n][b][T]) and (T-max(ST[n][b])) > RdSabs: # and T-
max(dST[N-nI][B]) > RdSabs :
                    ST[n][b] = ST[n][b] + [T]
                    if ST[n][b].count(0) == 1:
                        ST[n][b].remove(0)
```

```
        if P==1:
            if dLTP == 1:
                dLTPsub (n,b,W,Wm)
            if dOA == 1:
                dOAsub (n,b,TOH)
    # BSP
    if max(ST[n][b]) > 1 :
        y = T - max(ST[n][b])
        # Sodium spike
        if y > dNMDA:
            D[n][b][T]= KdS*(1+y)*G2**(-y-1)
        else:
            D[n][b][T] = KdS * 2 * G2**(-2)

    Ddabs[n][b][T] = D[n][b][T] + gR
    DEO[n][b][T] =  D[n][b][T] + E[n][b][T] + O[n][b][T] +gR  # sum D E O at branch

    # Somatic calculations
    E[n][0][T] = E[n][0][T] + E[n][b][T]*Upass         # value of D at soma
    D[n][0][T] = D[n][0][T] + D[n][b][T]*Uk            # value of summed E at soma
    Eabs[n][0][T] = E[n][0][T] + E[n][b][T]*Upass +gR   # absolute value of summed E at soma
    Dabs[n][0][T] = D[n][0][T] + D[n][b][T]*Uk +gR      # absolute value of D at soma
    b= b+1

# end while b
# Relative somatic refractorines
Ref = 0
if max(ST[n][0]) > 0:
    if  T > max(ST[n][0])+2:
        Ref = KRef*(T-max(ST[n][0]))*G3**(max(ST[n][0])-T)
if T > max(ST[n][0])+2:                              # after absolute refractoriness
    M[n][0][T] = R[n][0][T] + E[n][0][T] + D[n][0][T] + Ref
```

```
        # AP generation
        if (S[n][0][T] < M[n][0][T]) and T < (nT-3) and (T-max(ST[n][0])) > 2: #2 ms as absolute
refractoriness
          ST[n][0] = ST[n][0] + [T+1]                    # AP at this time point!
          M[n][0][T] = 30                            # AP potential
          M[n][0][T+1] = -1000                       # absolute somatic refractoriness
          M[n][0][T+2] = -1000                       # absolute somatic refractoriness
        if ST[n][0].count(0) > 0 and len(ST[n][0])>1:         # eliminate zero in ST
          ST[n][0].remove(0)
      # end n
    return(dLTP,dOA)


# ==============================================================================
def plot(P,b):

    Tplot = list(range(3,nT))

    for n in range(nI+1,nI+2):          # neurons to plot
      for b in range(0,13): #nB0):
        # encoding
        if P == 1:
          print("ENCODING - Membran currents:")
          if b == 0:
            print("Neuron", n, "soma")
          else:
            print("Neuron", n, "branch", b)

          plt.ylabel('MP [mV]')
          plt.xlabel('time [ms]')
          plt.grid(True)

          if b == 0:
```

```
        xmin, xmax, ymin, ymax = 3.0, nT, -75.0, -40.0
        plt.axis([xmin,xmax,ymin, ymax])
        plt.fill_between(Tplot, -75.0, -40.0, color='y',alpha = .1)
        plt.ylim(-75.0, -40.0)

        plt.plot(R[n][b],'k--', linewidth = 2.0, markersize=0)
        plt.plot(S[n][b], 'k--', linewidth = 2.0, markersize=0)
        #plt.plot(M[n][b],'k', linewidth = 1.0, markersize=0)
        plt.plot(Dabs[n][b],'b', linewidth = 1.0, markersize=0)
        plt.plot(Eabs[n][b],'r--', linewidth = 1.0, markersize=0)

    else:
        xmin, xmax, ymin, ymax = 3.0, nT, -75.0, -40.0
        plt.axis([xmin,xmax,ymin, ymax])
        plt.fill_between(Tplot, -75.0, -40.0, color='y',alpha = .1)
        plt.ylim(-75.0, -40.0)

        plt.plot(S[n][b], 'k--', linewidth = 2.0, markersize=0)
        plt.plot(R[n][b], 'k--',   linewidth = 2.0, markersize=0)
        plt.plot(Esabs[n][b][0],'r', linewidth = 1.0, markersize=0)
        plt.plot(Esabs[n][b][1],'r--',  linewidth = 1.0, markersize=0)
        plt.plot(Oabs[n][b],'g--',  linewidth = 2.0, markersize=0)
        plt.plot(Ddabs[n][b],'b', linewidth = 1.0, markersize=0)
        plt.plot(DEO[n][b],'k', linewidth = 1.0, markersize=0)

    plt.show()

if P == 2:
    print("RECALL - Membran currents:")
    if b == 0:
        print("Neuron", n, "soma")
    else:
```

```
    print("Neuron", n, "branch", b)

plt.ylabel('MP [mV]')
plt.xlabel('time [ms]')
plt.grid(True)

if b == 0:
    xmin, xmax, ymin, ymax = 3.0, nT, -75.0, -40.0
    plt.axis([xmin,xmax,ymin, ymax])
    plt.fill_between(Tplot, -75.0, -40.0, color='y',alpha = .1)
    plt.ylim(-75.0, -40.0)

    plt.plot(R[n][b],'k--', linewidth = 2.0, markersize=0)
    plt.plot(S[n][b], 'k--', linewidth = 2.0, markersize=0)
    plt.plot(R[n][b],'k--', linewidth = 2.0, markersize=0)
    plt.plot(S[n][b], 'k--', linewidth = 2.0, markersize=0)
    #plt.plot(M[n][b],'k', linewidth = 1.0, markersize=0)
    plt.plot(Dabs[n][b],'b', linewidth = 1.0, markersize=0)
    plt.plot(Eabs[n][b],'r--', linewidth = 1.0, markersize=0)

else:
    xmin, xmax, ymin, ymax = 3.0, nT, -75.0, -40.0
    plt.axis([xmin,xmax,ymin, ymax])
    plt.fill_between(Tplot, -75.0, -40.0, color='y',alpha = .1)
    plt.ylim(-75.0, -40.0)

    plt.plot(S[n][b], 'k--', linewidth = 2.0, markersize=0)
    plt.plot(R[n][b], 'k--',   linewidth = 2.0, markersize=0)
    plt.plot(Esabs[n][b][1],'r--',  linewidth = 1.0, markersize=0)
    plt.plot(Oabs[n][b],'g--',  linewidth = 2.0, markersize=0)
    plt.plot(Ddabs[n][b],'b', linewidth = 1.0, markersize=0)
    plt.plot(DEO[n][b],'k', linewidth = 1.0, markersize=0)
```

```
            plt.show()

    return(P,b)

# ================================================================================
def variables_begin():

    print("\n")
    print("A Model of Memory Linking Time to Space")
    print("Hubert LÃ¶ffler, Daya Shankar Gupta","\n")
    print("SIMULATION PARAMETERS:","\n")

    print ("nT:        ", nT)
    print("nN:         " ,nN)
    print ("nI:         ", nI)
    print ("dLTP:        ", dLTP, "       dPOA:      ", dOA)
    print("dGOPh:      ",dGOPh,"       TOPh:    ",dTOPh)
    print("GammaFq:   ",GammaFq,"     ThetaFq:   ", "{0:.2f}".format(ThetaFq))

    print("Kabs_s:     ",Kabs_s, "     Kabs_d:   ",Kabs_d,"     KRef:",KRef)
    print("KdS:        ",KdS, "     Upass:     ",Upass,"    Uk:     ",Uk)
    print("G1_EPSP:     ",G1, "     G2_dS:     ",G2,"     G3_Ref: ",G3)
    print("absRef_AP:   ",RSabs, "       absRef_dS: ",RdSabs,"       dNMDA:  ",dNMDA)
    print("nB0:        ",nB0, "       nSyn:      ",nSyn,"\n")

    print("Phases of branches of neuron[E]","\n",TOPh[3])
    print("\n")
    print("Connections of branches of neuronneuron[E]","\n",C[3])
    print("\n")
    print("Spike times of neuron[I]","\n",ST[1][0])
    print("Spike times of neuron[A]","\n",ST[2][0])
```

```
    print("\n")

    return

# ==============================================================================
def variables_end_E():

    print("\n")
    print("ENCODING Results:","\n")
    for n in range(nI+1,nN+1):
        print("APs of neuron[E]","\n",ST[n][0])
    print("\n")
    output(P,b)
    return

# ==============================================================================
def variables_end_R():

    print("\n")
    print("RECALL Results:","\n")
    for n in range(nI+1,nN+1):
        print("APs of neuron[E]","\n",ST[n][0])
    print("\n")
    output(P,b)
    return

# ==============================================================================
def output(P,b):

    print("Dendritic spikes at neuron[E]")
    for b in range(1,13):
        if ST[3][b][0] is not 0 :
```

```
            print("b[",b,"] T", ST[3][b])
    print("\n")
    for n in range(nI+1,nN+1):
       if P==1: print("Synaptic weights at branches of neuron[E]","\n",W[n])
    print("\n")
    return(P,b)

# ============================================================================
# MAIN  MAIN   MAIN   MAIN   MAIN   MAIN   MAIN   MAIN   MAIN   MAIN  MAIN   MAIN   MAIN
MAIN   MAIN   MAIN   MAIN   MAIN


# A Model of Memory Linking Time to Space
# Hubert LÃ¶ffler, Daya Shankar Gupta


# P: part of simulation: 1 = encoding; 0 = recall
# dLTP: learning by dendritic LTP at synapses from I
# dOA: learning by amplification of oscillation
# pltE: plot variables during encoding
# pltR: plot variables during recall
# pltR: plot variables during learning
# var_begin: print simulation parameters before simulation
# var_end: print simulation parameters after simulation


# nT: final time point of simulation (ms)
# nN: number of simulated neurons (1,2,3)
# nI: number of input neurons (1,2)


# dGOPh: duration of gamma-oscillation cycle
# dTOPh: duration of ttheta-oscillation cycle
# fTOPh: first theta-oscillastion phase


# nB0: number of branches
```

nSyn number of synapses, weights, masx-weights per branch

Kabs_d: k for EPSP at branches in f(EPSP)

Kabs_s: k for EPSP at soma in f(EPSP)

KRef: k for relative refracotory period

G1 constant related to EPSP

G2 constant related to dendritic spike

G3 constant related to refractory period

kdS: k for dendritic spike potential in f(dSP)

Upass: passive decay of branch potential to soma

Uk: branch strength

RSabs: absolute refractoryness of somatic sikes

RdSabs: absolute refractoriness of dendritic spikes

dNMDA: duration of NMDA-spike

gR: general resting potential at all neurons at soma and at all branches

gS: general somatic spiking threshold

dS: general dendritic spiking threshold

gTOH: general Theta-oscillation hight

gTOPh: general Theta oscillation phase

gTOFq: general Theta-oscillation frequency

TOHmax: enhanced oscillation hight

GammaFq: gamma-frequency of input

ThetaFq: theta-frequency general frequency of SMO

C: connections: (0,2): axons arriving from neuron 1 and 2

dW: weight of dendritic synapses

W: synaptic weight for EPSP caused by ariving AP (somatic and dendritic)

```
# Wm: maximal weightof dendritic synapses
# ST: spike times (somatic and dendritic)
# R: actual resting potential
# S: actual spiking threshold
# O: oscillation potential (0,13): 0 = at soma, 1-12 = at dendritic branches
# TOH: theta-oscillation hight (somatic and dendritic)
# TOFq: theta-oscillation frequency (somatic and dendritic)
# TOPh: theta-oscillation phase (somatic and dendritic)

# M: actual membran potential
# E: actual evoked potetial: 0 = soma; 1-12 = dendritic
# Eabs: absolute E
# O: actual SMO potential
# Oabs: absolute O
# D: dendritic spike potential
# Dabs: absolute D
# Ddabs: absolute dendritic spike potential
# DEO: branch spike potential  [= D+E+O]
# Es:  sum of all EPSPs at the branch)

# Ref: function for relative somatic refractorines
# n: neurons (0,nN); [1 = neuron I; 2 = neuron A; 3 = neuron E]
# b: dendritic branches (0,13) of neuron 3

nST = input("Write the number of randomly selected inpu spike trains:")
if nST == "":
  nST = 1
a = 1

while a <= int(nST):
   # choose dLTP or dOA as 1 for learning method
```

```
dLTP = 1                    # LTP of synapses by dendritic spikes [only from neuron 6 and only during encoding]
dOA = 0                     # oscillation amplification by dendritik spikes [only during encoding]
# choose pltE or/and pltR for ploting results of Encoding or/and results of Recall
pltE = 1                    # plot Encoding
pltR = 1                    # plot Recall
# choose var_begin or/and var_end for printing results of Encoding or/and results of Recall
var_begin = 1               # print simulation results by encoding
var_end = 1                 # print simulation results by recall

# ENCODING
P = 1                       # Part of simulation (Encoding = 1; Recall = 2)
# Read from cvs
d = open("NN_input_supplement.csv")
all = d.read()
d.close()
linelist = all.split(chr(10))

itemlist = linelist[1].split(";")
nT = int(itemlist[0])
nN = int(itemlist[1])
nI = int(itemlist[2])

itemlist = linelist[3].split(";")
dGOPh = int(itemlist[0])          # duration of Gamma-oscillation cycle
dTOPh = int(itemlist[1])          # duration of Theta-oscillation cycle
fTOPh = int(itemlist[2])          # first Theta-oscillastion phase

itemlist = linelist[5].split(";")
nB0 = int(itemlist[0])            # number of branches, necessary for one theta cycle +1
nSyn = int(itemlist[1])           # number of synapses, weight, masx-weights per branch
```

```
itemlist = linelist[7].split(";")
Kabs_s = float(itemlist[0])          # constant for somatic EPSP
Kabs_d  = float(itemlist[1])         # constant for dendritic EPSP
KRef = float(itemlist[2])            # constant for relative refracotory period

itemlist = linelist[9].split(";")
G1 = float(itemlist[0])              # constant related to EPSP
G2 = float(itemlist[1])              # constant related to dendritic spike
G3 = float(itemlist[2])              # constant related to refractory period

itemlist = linelist[11].split(";")
KdS = float(itemlist[0])             # constant for dendritic spike
Upass = float(itemlist[1])           # passive EPSP decay to soma
Uk = float(itemlist[2])              # branch strength

itemlist = linelist[13].split(";")
RSabs = int(itemlist[0])             # absolute refractoryness of somatic sikes
RdSabs = int(itemlist[1])            # absolute refractoriness of dendritic spikes
dNMDA = int(itemlist[2])             # duration of NMDA-spike

itemlist = linelist[15].split(";")
gR = float(itemlist[0])              # general resting potential at all neurons at soma and at all branches
gS = float(itemlist[1])              # general somatic spiking threshold
dS = float(itemlist[2])              # general dendritic spiking threshold
gTOH = float(itemlist[3])            # general Theta-oscillation hight
gTOPh= float(itemlist[4])            # general Theta oscillation phase
gTOFq = float(itemlist[5])           # general Theta-oscillation frequency
TOHmax = float(itemlist[6])          # enhanced oscillation hight

GammaFq = 1000/dGOPh                 # gamma-frequency of input
ThetaFq = 1000/dTOPh                 # theta-frequency general frequency of SMO
```

```
# Connections
C = [0]*(nN+1)
for n in range(nI+1,nN+1):
   C[n] =[0]*(nB0)
   for b in range(1,nB0):
      C[n][b]=[1,2]

# Synaptic weights of dendritic conections of neuron 3 from neuron 1 and 2
dW = [0,0,0]
W = [0]*(nN+1)
for n in range(nI+1,nN+1):
   W[n] =[0]*(nB0)
   for b in range(1,nB0):
      W[n][b]=[0.15,0.4]

# Maximal synaptic weights of dendritic conections of neuron 3 from neuron 1 and 2
Wm = [0]*(nN+1)
for n in range(nI+1,nN+1):
   Wm[n] =[0]*(nB0)
   for b in range(1,nB0):
      Wm[n][b]=[0.15,0.55]

# Preparing input spike times
ST = [0]*(nN+1)
for n in range(0,nI+1):ST[n]=[0]
for n in range(1,nN+1):ST[n]= [[0]]*nB0

# Random input spike trains from neuron 1
z=0
xx=0
for z in range(1,nI):
   x = random.randint(1,nB0-1)
```

```
    ST[z][0] = [0] * x
    y=0
    while y < x :
        xx = random.randint(1,nB0-1) * dGOPh
        if xx in ST[z][0]:
            x=x
        else:
            ST[z][0].append(xx)
            ST[z][0].remove(0)
            ST[z][0].sort()
            y = y + 1

# Input example reported in the article: for simulation this input: uncomment next line
ST[1][0] = [10,20,30,70,100,110]
#ST[1][0] = [20,100,110]

ST[2][0] = [10,20,30,40,50,60,70,80,90,100,110,120]



# Neuronal parameters
R = [0]*(nN+1)                    # resting potential
for n in range(nI+1,nN+1):
    R[n] =[0]*nB0
    for b in range(0,nB0):
        R[n][b]=[gR]*nT

S = [0]*(nN+1)                    # threshold potential
for n in range(nI+1,nN+1):
    S[n]=[0]*nB0
    for b in range(0,nB0):
        if b == 0:
            S[n][b] = [gS]*nT
```

```
        else:
            S[n][b] = [dS]*nT

dO = [0,0]                            # theta oscillation hight
TOH = [0]*(nN+1)
for n in range(nI+1,nN+1):
    TOH[n] =[gTOH]*nB0
    for b in range(0,nB0):
        TOH[n][b]=gTOH
        if b==0:TOH[n][b]=0

TOFq = [0]*(nN+1)                     # theta oscillation frequency
for n in range(nI+1,nN+1):
    TOFq[n] =[gTOFq]*nB0
    for b in range(0,nB0):
        TOFq[n][b]=gTOFq

TOPh = [0]*(nN+1)                     # theta oscillstion phase
for n in range(nI+1,nN+1):
    TOPh[n] =[gTOPh]
    for n in range(nI+1,nN+1):
        TOPh[n]=[gTOPh]*nB0
        TOPh[n]=[0]*nB0
        for b in range(0,nB0):
            TOPh[n][b] = fTOPh + dGOPh*b

# O, Oabs, E, Eabs, D, Dabs, Ddabs, DEO, M, Es[], Esabs[]
M = [0]*(nN+1)                        # membran potential
for n in range(nI+1,nN+1):
    M[n] =  [0]*nB0
    for b in range(0,nB0):
        M[n][b] = [0]*nT
```

```
        for t in range(0,nT):
            M[n][b][t] = 0.0

E = [0]*(nN+1)                          # summed EPSPs
for n in range(nI+1,nN+1):
    E[n] =  [0]*nB0
    for b in range(0,nB0):
        E[n][b] = [0]*nT
        for t in range(0,nT):
            E[n][b][t] = 0.0

Eabs = [0]*(nN+1)                       # summed EPSPs absolute
for n in range(nI+1,nN+1):
    Eabs[n] =  [0]*nB0
    for b in range(0,nB0):
        Eabs[n][b] = [0]*nT
        for t in range(0,nT):
            Eabs[n][b][t] = 0.0

O = [0]*(nN+1)                          # oscillation potential
for n in range(nI+1,nN+1):
    O[n] =  [0]*nB0
    for b in range(0,nB0):
        O[n][b] = [0]*nT
        for t in range(0,nT):
            O[n][b][t] = 0.0

Oabs = [0]*(nN+1)                       # oscillation potential absolute
for n in range(nI+1,nN+1):
    Oabs[n] =  [0]*nB0
    for b in range(0,nB0):
        Oabs[n][b] = [0]*nT
```

```
        for t in range(0,nT):
          Oabs[n][b][t] = 0.0

D = [0]*(nN+1)                         # dendritic spike potential
for n in range(nI+1,nN+1):
   D[n] =  [0]*nB0
   for b in range(0,nB0):
      D[n][b] = [0]*nT
      for t in range(0,nT):
         D[n][b][t] = 0.0

Dabs = [0]*(nN+1)                      # dendritic spike potential at soma absolute
for n in range(nI+1,nN+1):
   Dabs[n] =  [0]*nB0
   for b in range(0,nB0):
      Dabs[n][b] = [0]*nT
      for t in range(0,nT):
         Dabs[n][b][t] = gR

Ddabs = [0]*(nN+1)                     # dendritic spike potential at branch absolute
for n in range(nI+1,nN+1):
   Ddabs[n] =  [0]*nB0
   for b in range(0,nB0):
      Ddabs[n][b] = [0]*nT
      for t in range(0,nT):
         Ddabs[n][b][t] = 0.0

DEO = [0]*(nN+1)                       # branch spike potential
for n in range(nI+1,nN+1):
   DEO[n] =  [0]*nB0
   for b in range(0,nB0):
      DEO[n][b] = [0]*nT
```

```
    for t in range(0,nT):
      DEO[n][b][t] = 0.0

Es  = [0]*(nN+1)                      # EPSP per synapse
for n in range(nI+1,nN+1):
  Es[n] = [0]*nB0
  for b in range(0,nB0):
    Es[n][b] = [0]*nSyn
    for s in range(0,nSyn):
      Es[n][b][s] = [0]*nT
      for t in range(0,nT):
        Es[n][b][s][t]=0.0

Esabs  = [0]*(nN+1)                   # EPSP per synapse absolute
for n in range(nI+1,nN+1):
  Esabs[n] = [0]*nB0
  for b in range(0,nB0):
    Esabs[n][b] = [0]*nSyn
    for s in range(0,nSyn):
      Esabs[n][b][s] = [0]*nT
      for t in range(0,nT):
        Esabs[n][b][s][t] = gR

#  Simulation parameters for printing
if var_begin == 1 and a == 1:
  variables_begin()

simulation(dLTP,dOA)

if pltE==1:
  plot(P,b)
```

```
# Results after encoding
if var_begin == 1:
   variables_end_E()

#""""""""""""""""""""""""""""""""""""""""""""""""""""
# RECALL
P = 2
dLTP = 0   # 0 = no learning during recall
dOA = 0    # 0 = no oscillation amplification during recall

# Spike times of neurons 1 is set to 0
for n in range(1,nN+1):
   ST[n]=[[0]]*nB0

# Spike times of neuron 2 is set to bursting
ST[2][0] = [10,20,30,40,50,60,70,80,90,100,110,120] # input burst

# Reset variables M,E,Eabs,D,Dabs,Ddabs,DEO,Es,Esabs
M = [0]*(nN+1)                     # membran potential
for n in range(nI+1,nN+1):
   M[n] =  [0]*nB0
   for b in range(0,nB0):
      M[n][b] = [0]*nT
      for t in range(0,nT):
         M[n][b][t] = 0.0

E = [0]*(nN+1)                     # summed EPSPs
for n in range(nI+1,nN+1):
   E[n] =  [0]*nB0
   for b in range(0,nB0):
      E[n][b] = [0]*nT
      for t in range(0,nT):
```

```
      E[n][b][t] = 0.0

Eabs = [0]*(nN+1)                    # summed EPSPs
for n in range(nI+1,nN+1):
   Eabs[n] =  [0]*nB0
   for b in range(0,nB0):
      Eabs[n][b] = [0]*nT
      for t in range(0,nT):
         Eabs[n][b][t] = 0.0

D = [0]*(nN+1)                       # dendritic spike potential
for n in range(nI+1,nN+1):
   D[n] =  [0]*nB0
   for b in range(0,nB0):
      D[n][b] = [0]*nT
      for t in range(0,nT):
         D[n][b][t] = 0.0

Dabs = [0]*(nN+1)                    # dendritic spike potential
for n in range(nI+1,nN+1):
   Dabs[n] =  [0]*nB0
   for b in range(0,nB0):
      Dabs[n][b] = [0]*nT
      for t in range(0,nT):
         Dabs[n][b][t] = gR

Ddabs = [0]*(nN+1)                   # dendritic spike potential at branch absolute
for n in range(nI+1,nN+1):
   Ddabs[n] =  [0]*nB0
   for b in range(0,nB0):
      Ddabs[n][b] = [0]*nT
      for t in range(0,nT):
```

```
         Ddabs[n][b][t] = 0.0

DEO = [0]*(nN+1)                    # branch spike potential
for n in range(nI+1,nN+1):
   DEO[n] =  [0]*nB0
   for b in range(0,nB0):
      DEO[n][b] = [0]*nT
      for t in range(0,nT):
         DEO[n][b][t] = 0.0

Es  = [0]*(nN+1)                    # EPSP per synapse
for n in range(nI+1,nN+1):
   Es[n] = [0]*nB0
   for b in range(0,nB0):
      Es[n][b] = [0]*nSyn
      for s in range(0,nSyn):
         Es[n][b][s] = [0]*nT
         for t in range(0,nT):
            Es[n][b][s][t]=0.0

Esabs  = [0]*(nN+1)                 # EPSP per synapse absolute
for n in range(nI+1,nN+1):
   Esabs[n] = [0]*nB0
   for b in range(0,nB0):
      Esabs[n][b] = [0]*nSyn
      for s in range(0,nSyn):
         Esabs[n][b][s] = [0]*nT
         for t in range(0,nT):
            Esabs[n][b][s][t] = gR

simulation(dLTP,dOA)
```

```
# Results after recall
if var_end == 1:
    variables_end_R()


if pltR==1:
    plot(P,b)


a = a + 1
# end a
```
