# Supplementary figures and images for: A Model of Memory Linking Time to Space
Source: Front Comput Neurosci. 2020 Jul 8;14:60. doi: 10.3389/fncom.2020.00060 (PMC7360808; doi:10.3389/fncom.2020.00060)

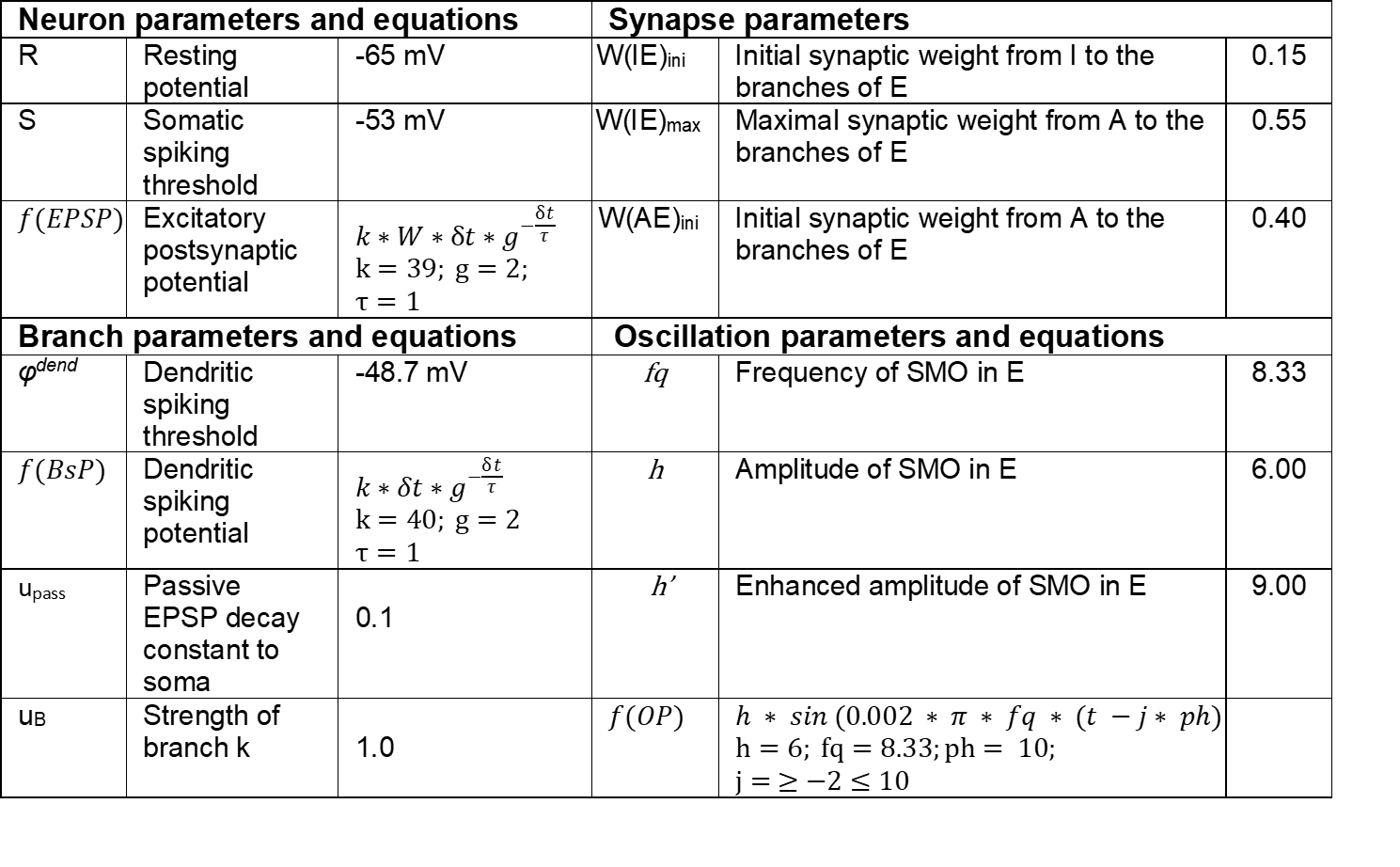

Supplement: Supplementary file 2 [file Image_1.TIF]
