# Supplementary material for: A Model of Memory Linking Time to Space
Source: Front Comput Neurosci. 2020 Jul 8;14:60. doi: 10.3389/fncom.2020.00060 (PMC7360808; doi:10.3389/fncom.2020.00060)
Supplement: Supplementary file 3 [file Table_1.pdf]

Table 1: Parameters of neurons, synapses, branches, and SMOs.

**Neuron parameters and equations**		
R	Resting potential	- 65 mV
S	Somatic spiking threshold	- 53 mV
f(EPSP)	Excitatory postsynaptic potential [Formula (2)]	k = 39; g = 2; τ = 1
**Branch parameters and equations**		
φ^{dend}	Dendritic spiking threshold	- 48.7 mV
f(BsP)	Dendritic spiking potential [Formula (3)]	k = 40; g = 2; τ = 1
u_{pass}	Passive EPSP decay constant to soma	0.1
uB	Strength of branch	1.0
**Synapse parameters**		
$W(IE)_{ini}$	Initial synaptic weight from I to E	0.15
$W(IE)_{max}$	Maximal synaptic weight from A to E	0.55
$W(AE)_{ini}$	Initial synaptic weight from A to E 0.40	
**SMO parameters and equations**		
fq	Frequency of SMO in E	8.33
h	Amplitude of SMO in E	6.00
h’	Enhanced amplitude of SMO in E	9.00
f(OP)	Osciillation potential [Formula (1)]	h = 6; fq = 8.33; ph = 10; j = ≥ -2 ≤ 10
