# Supplementary material for: A Model of Memory Linking Time to Space
Source: Front Comput Neurosci. 2020 Jul 8;14:60. doi: 10.3389/fncom.2020.00060 (PMC7360808; doi:10.3389/fncom.2020.00060)
Supplement: Supplementary file 4 [file Table_2.pdf]

```
nT;nN;nI;;;;
126;3;2;;;;
dGOPh;dTOPh;fTOPh;;;;
10;120;-20;;;;
nb0;nSyn;;;;;
13;2;;;;;
Kabs_s;Kabs_d;KRef;;;;
10;39;-250;;;;
G1;G2;G3;;;;
2;2;2.700;;;;
KdS;Upass;Uk;;;;
40;0.08;1.0;;;;
RSabs;RdSabs;dNMDA;;;;
2;5;0;;;;
gR;gS;dS;gTOH;gTOPh;dTOFq;TOHmax
-65;-53;-48.7;6;0;8.333;9
```
